# Effectiveness of psychoeducational intervention in promoting post-stroke self-care: a systematic literature review

**DOI:** 10.3389/fresc.2025.1569526

**Published:** 2025-06-06

**Authors:** Victor Nhime Nungulo, Mauer Gonçalves, Maria Adriana Henriques, Cristina Lavareda Baixinho

**Affiliations:** ^1^ Faculty of Medicine of Huambo, José Eduardo dos Santos University, Huambo, Angola; ^2^ Centre for Research, Innovation and Development in Nursing, Lisbon (CIDNUR), Lisbon, Portugal; ^3^ Centre for Advanced Studies in Medical Education and Training, Faculty of Medicine, Agostinho Neto University, Luanda, Angola; ^4^ School of Nursing, Lisbon, Portugal

**Keywords:** stroke, promotion, psychoeducational intervention, self-care, systematic review

## Abstract

**Background:**

Stroke is one of the main causes of morbidity and mortality in the world, and sequelae include physical disability, difficulties with independence for self-care, and loss of mobility and quality of life.

**Objective:**

this study was to summarize the evidence about the effectiveness of psychoeducational interventions in promoting post-stroke self-care.

**Methods:**

A systematic literature review was carried out per the Cochrane recommendations. The following databases were included: MEDLINE (via PubMed), Scopus, CINAHL Complete, Cochrane, JBI, and B-on. The study as performed between December 2023 and February 2024, according to the eligibility criteria, by two independent reviewers. The bibliographic sample was evaluated for risk of bias using RoB 2, only clinical trials were included.

**Results:**

The seven articles selected, from 2019 to 2024 evaluated education to promote care and self-care; rehabilitation programmes with physical exercise; management of stress, depression and anxiety; and symptom management. Interventions relating to awareness and knowledge about post-stroke, development of healthy behavior and lifestyle reinforcement of self-care capacity.

**Conclusions:**

This literature review found that in some studies the implementation of psychoeducational interventions improves the knowledge, independence and self-care of this population and their families, although not all of them were equally effective. The results of the articles reinforce that psychoeducational interventions may increase functional independence and the ability to carry out activities of daily living and improve health and quality of life.

**Systematic Review Registration:**

https://www.crd.york.ac.uk/PROSPERO/view/CRD42023483087, PROSPERO CRD42023483087.

## 1 Background

Stroke is the second leading cause of mortality in developing countries and one of the leading causes of temporary or permanent physical disability in the adult and older adult population (1, 2). About 75% of stroke patients show disabilities in independent daily life abilities (1). Although during hospitalization these patients have rehabilitation, usually with a positive impact on the functional capacity (1–3), this approach has limitations due to systematic discharge preparation, insufficient community integration procedures, lack of information on community services, and poor access to community services (1).

The aforementioned factors, in addition to the lack of continuity in the rehabilitation program started in hospital, the caregivers' difficulties in ensuring the satisfaction of activities of daily living and the lack of preparation of formal caregivers and home support services to promote the independence of these people, contribute to increased immobility, difficulties in self-care, changes in social support, depression, isolation and loss of quality of life (1, 3).

A systematic review (SR) that aimed to summarize evidence on longer-term unmet needs perceived by stroke survivors concluded that they can be grouped into three categories: body functions, activities/participation and environmental needs (4). Unmet body function needs included mainly psychosocial or cognitive problems, fatigue, and pain, while unmet needs regarding activities/participation concerned mainly mobility, leisure time, and employment. In terms of unmet environmental needs, the most commonly reported was the unmet needs for services (4).

Secondary post-stroke sequelae are associated with difficulties of healthcare systems with guaranteeing continuity of care and long-term support to these patients related to their physical condition, self-care, and the adoption of behaviors that ensure a healthy lifestyle, emotional balance, and participation in social life (3, 4).

The results of the studies were in agreement in affirming that existing rehabilitation provision for stroke survivors does not address their long-term needs (4–6). After a stroke, most patients require rehabilitation for neurological and neuropsychological sequelae, 70% do not return to work, and 30% need support to walk. Post-discharge nursing care and professional guidance to promote self-care are crucial for the performance of activities of daily living (7).

To best assist patients' recovery, innovative research has sought to develop and evaluate behavioral approaches, identify and refine synergistic approaches that augment response to behavioral therapy, and integrate technology where appropriate, particularly to introduce and titrate real-world complexity and improve the overall experience of therapy (8).

In terms of behavioral approaches, psychosocial and psychoeducational interventions can promote post-stroke adaptation and help people better manage the rehabilitation process, promoting self-care management, education, and physical exercise, which have a positive impact on the quality of life of these people and their families (8, 9). This, in turn, reduces the economic impact on health systems related to the treatment of sequelae, complications, and readmissions (10).

Psychoeducation is increasingly recognized for its value in facilitating adaptation to a chronic disease diagnosis (11), because it empowers people and their families with the knowledge they need about the disease, enabling them to self-manage their health/disease processes, manage the therapeutic regimen, and promote independence (12). It also enables families to address a range of issues associated with stroke support needs and transition to caregiving roles (13–15), with positive effects on mood, stress, anxiety, and depression (16) and in promoting self-care (17).

One study that tested the effectiveness of a self-management intervention for stroke patients compared to usual care concluded that the intervention was effective in improving health and quality of daily life and suggested that psychoeducational interventions based on problem-solving and setting individual goals can improve stroke survivors' self-management skills (18).

A preliminary review did not identify any systematic reviews evaluating the effectiveness of psychoeducational interventions to promote selfcare after stroke.

The objective of this systematic literature review was to summarize the evidence on the effectiveness of psychoeducational interventions in promoting self-care in stroke patients.

## 2 Materials and methods

### 2.1 Study design

To meet the objective of the study, we opted for a systematic review of effectiveness, according to the Cochrane protocol (19) for this type of review.

The protocol of the systematic review was submitted to the PROSPERO platform with the registration number CRD42023483087.

### 2.2 Eligibility criteria

The inclusion and exclusion criteria of the studies were defined based on the research question “What is the effectiveness of psychoeducational interventions for the promotion of self-care in stroke patients?”, structured according to the acronym PICOS (Table 1).

**Table 1:** Eligibility criteria, 2024.

PICOS	Inclusion criteria	Exclusion criteria
Participants	Adults and older adults Studies about stroke survivors and their caregivers	Adolescents Institutionalized patients
Interventions	Psychoeducational interventions (that involve some therapeutic or skills development)	Passive educational activities, such as handing out leaflets or discharge training sessions for discharge without engagement by the patients or their families.
Comparator	Usual care	—
Outcomes	Self-care Performing activities of daily living Degree of dependence	—
Study design	Clinical trials	Study protocols; systematic reviews; observational studies Qualitative studies; editorials; grey literature.

PICOS, Participants, Interventions, Comparator, Outcomes, Study design.

The definition of psychoeducational intervention is complex because as a therapeutic intervention it envolves psychological and educational components (13) that can provide information and support to cope with the physical and emotional effects of a health condition (16). In these systematic review the intervention must have this two compoments, involving therapeutic or skills development in patients and their caregivers.

A time limit of 5 years (2019–2024) has been set to obtain recent studies. A full text filter was applied, and only publications in English, Spanish, French or Portuguese were included.

### 2.3 Data collection

The review included the following databases: MEDLINE (via PubMed), Scopus, CINAHL Complete, Cochrane, JBI, and B-on. Natural language and database-specific descriptors were used for each database. Table 2 shows the search strategy used with MEDLINE.

**Table 2:** Search strategy used in the MEDLINE (via PubMed), 2024.

(((((((((((((((Adult[Title/Abstract]) OR (and your adult[Title/Abstract])) OR (“middle age[Title/Abstract])) OR (older adult[Title/Abstract])) OR (act*[Title/Abstract])) OR (area*[Title/Abstract])) OR (older person[Title/Abstract])) OR (“adult"[MeSH Terms])) OR (for adult, frail older[MeSH Terms])) OR (adults, frail older[MeSH Terms]) NOT (“adolescent"[MeSH Terms]) NOT (“children[MeSH Terms]) AND ((ffrft[Filter]) AND (english[Filter] OR french[Filter] OR English[Filter] OR spanish[Filter]) AND (alladult[Filter] OR youngadult[Filter] OR adult[Filter] OR middleagedaged[Filter] OR middleaged[Filter] OR aged[Filter] OR 80andover[Filter]) AND (&) 2019:the year 2025[pdat]))), AND (((((((((((((((((((((stroke[Title/Abstract])) OR (cerebral stroke[Title/Abstract])) OR (life after stroke[Title/Abstract])) OR (post-stroke[Title/Abstract])) OR (stroke management[Title/Abstract])) OR post-stroke[Title/Abstract])) OR follow-up care for stroke[Title/Abstract])) OR (post-stroke care[Title/Abstract])) OR (Long-term stroke management[Title/Abstract])) OR (acute stroke[MeSH Terms])) OR (acute strokes[MeSH Terms])) OR (cerebral stroke[MeSH Terms])) OR (cerebral strokes[MeSH Terms]) AND ((ffrft[Filter]) AND (english[Filter] OR french[Filter] OR English[Filter] OR spanish[Filter]) AND (alladult[Filter] OR youngadult[Filter] OR adult[Filter] OR middleagedaged[Filter] OR middleaged[Filter] OR aged[Filter] OR 80andover[Filter]) AND (&) 2019:2024[pdat])))) AND (((((((((((((((((Psychoeduc*[Title/Abstract]) OR (intervention-economic*[Title/Abstract])) OR (Psychoeducational intervent*[Title/Abstract])) OR (Psychoeducation intervent*[Title/Abstract])) OR (Psycho-education[Title/Abstract])) OR (Psycho-education[Title/Abstract])) OR (Psychoeducational p*[Title/Abstract])) OR (Psychoeducation p*[Title/Abstract])) OR (Active education[Title/Abstract])) OR (alternatively-psychoedc*[Title/Abstract])) OR (behavioral therapy[Title/Abstract])) OR (results[Title/Abstract])) OR (Engaging[Title/Abstract])) OR (“education"[MeSH Terms])) OR (activities, education[MeSH Terms])) OR (collaboration, education[MeSH Terms])) AND ((ffrft[Filter]) AND (english[Filter] OR french[Filter] OR English[Filter] OR spanish[Filter]) AND (alladult[Filter] OR youngadult[Filter] OR adult[Filter] OR middleagedaged[Filter] OR middleaged[Filter] OR aged[Filter] OR 80andover[Filter]) AND (&) 2019:the year 2025[pdat])))) AND ((((((((selfcare[Title/Abstract]) OR (self-care[Title/Abstract])) OR (independence, [Title/Abstract])) OR (activities of daily living[Title/Abstract])) OR (ADLs[Title/Abstract])) OR (autonomy[Title/Abstract])) OR (“self-care[MeSH Terms])) OR (activities of daily living[MeSH Terms]) AND ((ffrft[Filter]) AND (english[Filter] OR french[Filter] OR English[Filter] OR spanish[Filter]) AND (alladult[Filter] OR youngadult[Filter] OR an adult[Filter] OR middleagedaged[Filter] OR middleaged[Filter] OR aged[Filter] OR 80andover[Filter]) AND (&) 2019:2024[pdat])))) AND ((((RCT[Title/Abstract])) OR (prospective randomised controlled trials[Title/Abstract])) OR (“randomized controlled trials on the topic"[MeSH Terms]) AND ((ffrft[Filter]) AND (english[Filter] OR french[Filter] OR English[Filter] OR spanish[Filter]) AND (alladult[Filter] OR youngadult[Filter] OR adult[Filter] OR middleagedaged[Filter] OR middleaged[Filter] OR aged[Filter] OR 80andover[Filter]) AND (&) 2019:2024[pdat])))Filters: a Free, full-text, English, French, Portuguese, Spanish, Adult: 19 + years, Young Adult: 19–24 years, Adult: 19–44 years, the Middle Aged + Aged: over 45 + years The Middle Aged: 45–64 years, Aged: 65 + years, 80 and over: 80 + years.

Data collection was carried out between December 2023 and February 2024 and updated in November 2024. The articles identified in the different databases were exported to the Rayyan® platform. After the elimination of duplicates, two reviewers independently screened the articles. A third reviewer was used to decide cases of non-consensus and omissions.

### 2.4 Data processing and analysis

After the selection of these articles, a form was created to guarantee efficient data management. The following information was extracted: author of study, year of publication and country of the study, study objective, study type, participants, intervention, results, and conclusions.

### 2.5 Risk of bias assessment

The risk of bias assessment of the included studies was conducted by the team of researchers independently using the RoB 2 Tool (Revised Cochrane risk-of-bias tool for randomized trials) (19). Disagreements among reviewers were discussed and resolved by the third reviewer.

### 2.6 Ethical considerations

Given that this study was a comprehensive literature review, it did not require the approval of an ethics committee. The recommendations of scientific ethics were followed, ensuring the adequate referencing of the articles included in the SLR and in the preparation of the article.

## 3 Results

**Figure 1:**
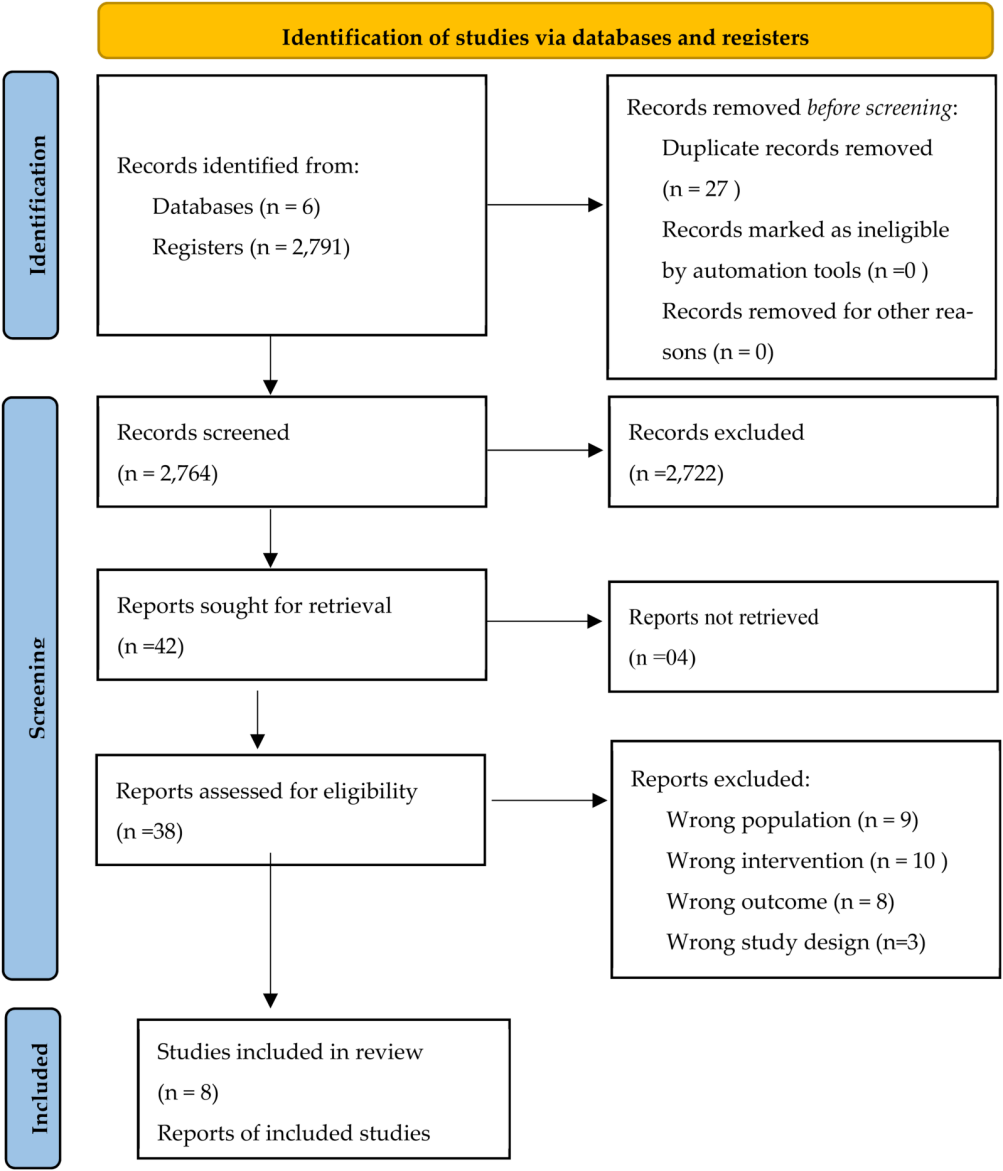
PRISMA 2020 flow diagram (20).

A total of 2660 articles was identified in the different databases, of which 27 were duplicates. Based on the analysis of titles, abstracts, and keywords, 38 articles were included for full reading, of which 30 were excluded because they did not meet the eligibility criteria (Figure 1). The final bibliographical sample includes 8 articles (10, 17, 21–26).

The articles had a heterogeneous geographical distribution: 1 was carried out in the United States (26); 2 in Australia (17, 21); 1 in Egypt (23); 1 in Turkey (10); 1 in Indonesia (24), 1 in Hong Kong (25) and 1 in China (22).

The eight clinical trials included (Table 3) identified psychoeducational interventions and their results. Different studies measured different outcomes, which enabled an evaluation of the impact of interventions on education, self-care promotion, rehabilitation, stress management, depression, anxiety, symptom management, functional independence, ability to perform activities of daily living, and quality of life.

**Table 3:** Studies included in the bibliographic sample.

Study/Year/Country	Objective	Participants	Intervention implemented	Results and conclusions
(Middleton et al. 2019) (21)/Australia	To assess the effectiveness of an intervention to improve the screening, treatment, and transfer of post-acute stroke patients admitted to the hospital.	Twenty-six (13 intervention and 13 control)	The intervention consisted of education sessions and the distribution of materials by the nurse researcher. Sessions were audio recorded to ensure accurate documentation of barriers and facilitators to inform site-specific action plans. the control group did not receive the clinical protocols or additional support.	This evidence-and theory-based implementation trial, previously effective in stroke units, did not change patient outcomes or physician behavior.
(Zhou et al. 2019) (22)/China	To determine the effectiveness of a new model of nurse-led and caregiver-provided stroke rehabilitation in rural China.	Stroke patients and caregivers in rural China (intervention *n* = 118; control (*n* = 128)	The new intervention was multifaceted and was based on a task-shifting/training-the-trainers model, supported by a custom-designed smartphone application, where patients and caregivers received evidence-based in-hospital education and stroke rehabilitation training (focus on mobility, self-care, and toileting), delivered by trained nurses before hospital discharge, and 3 post-discharge support telephone calls.	A new digitally supported, nurse-led, caregiver-delivered stroke rehabilitation program failed to improve patients' physical post-stroke functioning in rural China.
(Minshall et al. 2020) (17) Australia	To evaluate the effectiveness of a new psychosocial intervention aimed at improving health outcomes.	173 participants were recruited in a community-referred hospital services. (control: 39 stroke survivors and 40 caregivers; intervention: 50 patients and 44 caregivers)	The Intervention Group of post-stroke patients received personalised psychosocial support, support from a psychologist through a structured exercise book, educational information, a self-management plan, compliance with drug therapy and health plans.	This personalized psychosocial intervention resulted in significant improvement in caregiving satisfaction at 6 months, but with no other outcomes.
(Elsheikh et al. 2022) (23)/ Egypt	To evaluate the effectiveness of a multidimensional intervention adapted to reduce the burden of caregivers/family members of stroke survivors.	110 caregivers aged ≥18 years who cared for a survivor within 6 months of stroke, intervention group (IG; *n* = 55) and control group (CG; *n* = 55)	Psychoeducational intervention, skills development and peer support aimed at the needs of caregivers of stroke survivors.	Key findings showed that participants in the IG did not see an improvement in key outcomes. However, improvement in the psychological and social domains may be attributed to this intervention.
(Rasyid et al. 2023) (24)/ Indonesia	To test the effect of self-care stroke education (SSE) on changes in self-efficacy, self-care, and modification of risk factors.	120 patients. A total of 120 patients (intervention *n* = 60; standard care *n* = 60) were randomized	To use a visual-based educational model emphasizing concrete examples of self-care and increasing self-efficacy, such as the type of food consumed and a recommended exercise schedule.	In the 1st month, the intervention group showed a significant change in self-care (4.56 [95% CI: 0.57, 8.56]), self-efficacy (4.95 [95% CI: 0.84, 9.06]), and stroke risk (−2.33 [95% CI:−3.19, −1.47]) compared to the control group. In the 3rd month, the intervention group also showed a significant change in self-care (19.28 [95% CI: 16.01, 22.56]), self-efficacy (19.95 [95% CI: 16.61, 23.28]), and stroke risk (−3.83 [95% CI: −4.65, −3.01]) compared to the control group.
(Mou, et al. 2023) (25)/ Hong Kong	To examine the effects of a family-focused dyadic psychoeducational intervention on the functional and psychosocial outcomes of stroke survivors and family caregivers.	81 dyads (survivors and their family caregivers) in each group	The intervention was designed to enhance resources and perceptions for stroke dyads via three key components: information provision, psychological support, and emotional and behavioural regulation	Participants in the psychoeducation group revealed significantly greater reductions on caregiver burden than the control group at T_1_ (*β* = −6.01, *p* = 0.026) and T_2_ (β = −6.73, *p* = 0.039). In addition, the intervention demonstrated significantly greater improvements on caregiving competence (*β* = 0.98, *p* = 0.013; *β* = 1.58, *p* < 0.001), survivors' depressive symptoms (*β* = −1.56, *p* = 0.007; *β* = −2.06, *p* = 0.005), and dyadic relationship (*β* = 0.26, *p* = 0.012; *β* = 0.27, *p* = 0.022) at T_1_ and T_2_, as well as on survivor coping at T_2_ (*β* = 6.73, *p* = 0.008).
(Bal & Koç, 2024) (10)/Turkey	To determine the effect of technology-based health promotion training on activities of daily living, quality of life, and self-care of stroke survivors.	Was 70 patients (35 in the intervention group and 35 in the control group)	Intervention with education by telephone and follow-up care based on the theory of self-care.	When compared to the control group after the training session, there was a statistically significant difference in the intervention group's mean scores on the Stroke-Specific Quality of Life Scale (and the Exercise of Self-Care Agency Scale. Training interventions led to enhanced awareness and knowledge about stroke among the intervention group. They also fostered the development of healthier lifestyle behaviors and bolstered both self-care abilities and quality of life.
(Deijle et al. 2024) (26)/ USA	To investigate the effect of a physical exercise intervention on cognitive functioning after the intervention in people with TIA or minor stroke.	Patients with TIA or minor stroke were randomly allocated to an intervention group receiving a one-year exercise intervention (*n* *=* 60) or usual care (*n* *=* 59).	The 1-year MoveIT exercise intervention consisted of a 12-week physical fitness group exercise programme, followed by counselling sessions every 3 months for the remaining 9 months.	On average, the intervention group showed significant improvement in executive functioning when compared to the control group. The data showed that a one-year intervention of physical exercise significantly improved executive functioning over time compared to usual outcomes.

A risk of bias assessment was performed for each of the studies by applying the RoB 2 Tool (Table 4). It should be noted that there were concerns about bias in three studies (10, 24, 26).

**Table 4:** Risk of bias assessment of randomized controlled clinical trials ROB.2.0.

Study	D1	D2	D3	D4	D5	Overall
Middleton et al. (21)	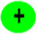	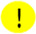	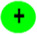	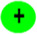	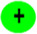	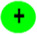
Zhou et al. (22)	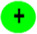	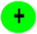	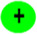	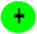	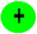	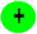
Minshall et al. (17)	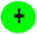	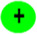	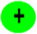	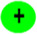	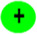	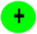
Elsheikh et al. (23)	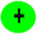	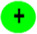	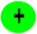	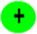	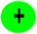	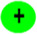
Rasyid et al. (24)	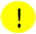	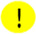	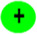	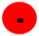	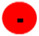	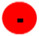
Mou et al. (25)	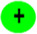	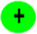	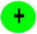	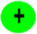	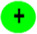	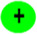
Bal & Koç (10)	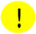	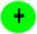	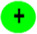	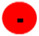	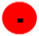	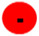
Deijle et al. (26)	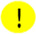	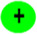	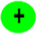	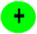	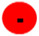	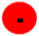

D1, bias arising from the randomization process; D2, bias due to deviations from intended interventions; D3, bias due to missing outcome data; D4, bias in the measurement of the outcome; D5, bias in the selection of the reported result.

Table 5 shows the types of interventions performed in each study and which are related to the objective of this SLR.

**Table 5:** Types of interventions.

Study	Type of intervention						
	Physical Exercise	Occupational Therapy	Education for self-care	Rehabilitation program	Capacity-building for activities of daily living.	Capacity-building for therapeutic manage-ment	Support and counseling
(10)			X	X	X		X
(17)			X	X			X
(21)			X				X
(22)		X	X	X			X
(23)			X				
(24)	X		X	X	X	X	X
(25)			X				X
(26)	X		X	X			X

The studies used a combination of two or more interventions to promote self-care. Education for selfcare in stroke survivors (10, 17, 21–26), was combined with the promotion of physical exercise (24, 26), occupational therapy (22), self-management of the therapeutic regimen (24), rehabilitation programs (10, 17, 22, 24, 26), training in the use of utensils (10, 24), and support and counseling (10, 17, 21, 22, 24–26). A meta-analysis could not be performed due to the heterogeneity of the interventions and outcomes. Even the same outcome, for instance, quality of life, was measure as a primary outcome (17) or as a secondary outcome (21–23).

The primary outcomes used to evaluate the effect of the intervention on patients were dependency (10, 21, 22), self-care (24), death, 90-day posthospital admission (21), functioning (25), cognitive functioning (26), quality of life (10, 17), self-efficacy (17, 24) and stroke risk scores after 1 and 3 months of discharge (24). The secondary outcomes were depression and anxiety symptoms, coping, disease perception, and personalized psychological and psychosocial support, social and work adjustment (17), 90-day functional dependency, health status (21), mobility and mood (21).

For caregivers the primary outcomes evaluated were: caregiver burden (17, 22, 23, 25), and caregiver satisfaction (17). The secondary outcomes included caregiving competence, dyads' coping, depressive and anxiety symptoms, family functioning, dyadic relationship, and caregiving-related injury (25).

## 4 Discussion

The seven studies included in this SLR show that psychoeducational interventions can have an impact on post-stroke self-care, mobility and functional capacity, independence in carrying out activities of daily living, self-management of therapeutic regimens, and the quality of life of these patients and their families (10, 17, 21–24, 26). Although the concerns with the risk of vies of three studies (10, 24, 26), they were included in this SR. In future studies, the implementation of these interventions, alone or in association, should be considered.

All the studies implemented interventios for the education for self-care. As state by several authors self-care needs are a major problem among stroke patients (1, 2, 25, 27) and nurses can support them through interventions such as education, with a positive change in their attitude and emphasis on their remaining abilities (27), but in clinical settings there are some barriers to the efficient teaching of patients such as the low proportion of the nurses to patients, busy and exhausting work schedules, lack of time for patient education and absence of caregivers (2, 27).

The association between education and rehabilitation programmes maintained over time has a positive effect on functionality, prevention of complications and patient satisfaction (10, 17, 22, 24, 26). These results corroborate those of other studies that have concluded that patients who adhere to rehabilitation and education programs have fewer depressive symptoms and have better self-care capacity and quality of life in comparison to those with greater difficulties in adherence (28, 29).

Rehabilitation programs that improve muscle strength, balance, gait ability, and motor coordination contribute to the promotion of self-care (30). Psychoeducation interventions are important to ensure the adherence of users to these programs, which have to be maintained over time to have an impact on functionality (17, 22, 26). It should be noted that a study that had as its primary aim testing the hypothesis that the use of psychoeducation leads to improved quality of life in patients after stroke concluded that IG patients experienced statistically greater improvements in quality of life than CG patients (*p* = 0.0005) (31).

This type of intervention is cost-effective and relatively easy to implement (16), and its use is recommended to introduce multidisciplinary and multicomponent interventions in community-based follow-up care, which can be initiated in the hospital, but which, due to the difficulties of these people and their families, justify their continuation in communities (23, 32).

The involvement of the family caregiver in the preparation of discharge is important to provide information, develop coping ability for helping in selfcare and reinforce the in-hospital intervention effects (8, 25). Planing discharge and give support during the first weeks at home allows to increase knowledge on stroke recovery and care, enhance coping abilities for challenges/difficulties in stroke selfcare and recovery, provide emotional support, and improve communication and relationships with their counterparts (25).This is due to its potential to improve the skills of patients and their families in order for them to understand the course of the disease, manage signs and symptoms, and be able to develop coping strategies (33).

An SLR showed the impact of incorporating programs with a focus on self-care and self-management on mitigating secondary risk in stroke survivors, particularly in those with uncontrolled hypertension (33). Moreover, psychoeducational programs have positive effects in reducing psychological distress in stroke patients (14) and family caregiver burden (23, 25, 32).

The results support the recommendation that health professionals, particularly nurses (25), introduce psychoeducational interventions in the follow-up care of stroke patients, possibly using digital health strategies (10, 24).

Future studies should associate the impact of this type of intervention on the control of common complications in this population, such as immobility syndrome and falling (33–36), given that reduced mobility of the lower limbs, attributed to the sequelae of stroke, leads to a vicious circle of sedentary behavior, atrophy, ankyloses and joint stiffness due to disuse, fear of falling, and falls (16, 31).

The self-care of stroke patients does not take place in isolation; it involves several actors, including families and other informal caregivers, who face daily doubts, concerns, and fears related to patients' health condition and how to support them in self-care. Furthermore, psychoeducational interventions have been shown to have positive results in solving family problems, and improving family communication and patient care (13), contributing to patient self-care.

### 4.1 Study limitations

Disparities were found in the identified (eligible) studies in relation to the interventions, so it was not possible to perform a meta-analysis. Studies whose results did establish an association between the intervention and the outcomes, in addition to the bias identified in three studies, limit recommendations for clinical practice. Nevertheless, there are studies that support recommendations for clinical practice and research.The search strategy was limited to Portuguese, English, Spanish and Frenchonly fulltext papers; studies conducted in other languages that answered t he research question may have been excluded.

## 5 Conclusions

The present study answered the research question about the effectiveness of psychoeducational interventions in the promotion of post-stroke self-care. The use of these interventions in association with the promotion of physical exercise, occupational therapy, self-management of therapeutic regimens, rehabilitation programs, training in the use of utensils, and support and counseling for patients and their families is safe and has an impact on patients and families in terms of self-care, increased functional independence, and reduced depression, anxiety, and post-stroke suffering.

The reduced number of studies included in this review and their heterogeneity in terms of interventions, number of participants, and measured outcomes allows the recommendation to new studies to increase the evidence about the effectiveness of the different interventions used in psychoeducation and their association with others, namely rehabilitation programs.

## Data Availability

The original contributions presented in the study are included in the article/Supplementary Material, further inquiries can be directed to the corresponding author.
